# P-1157. Clinical and Economic Outcome of Drugs Used in the Treatment of Pneumonia in Pediatric Population in a Tertiary Care Hospital in India-A Pharmacoeconomic Analysis

**DOI:** 10.1093/ofid/ofae631.1343

**Published:** 2025-01-29

**Authors:** Jameskutty Joseph

**Affiliations:** St. Joseph's College of Pharmacy, Chengalam, Kerala, India

## Abstract

**Background:**

Antibiotic therapy expenses for treating pneumonia in hospitalized pediatric patients can substantially impact healthcare expenditures. We conducted a longitudinal study to investigate prescription appropriateness, predictors of treatment patterns, and key outcomes—readmission rates, length of hospital stay (LOS), and direct and indirect costs associated with diagnosing and treating community-acquired pneumonia in pediatric patients admitted to a tertiary care hospital in India.

**Methods:**

We observed 180 children diagnosed with pneumonia and receiving antibiotic treatment upon admission to the pediatric inpatient department. We collected demographic data and prescription details, calculating direct and indirect costs. Multivariate regression analysis was employed to identify predictors of LOS and costs, while one-way ANOVA was utilized to assess cost disparities among treatment groups.

**Results:**

Among all pneumonia admissions, mild-to-moderate cases accounted for 78.1% of cases; 66% (119 out of 180) of children had comorbid conditions alongside pneumonia. Beta-lactam antibiotics constituted the majority of prescriptions (54%), followed by aminoglycosides (20%) and macrolides (12%). Parenteral antibiotics were administered in 62.2% of cases, and switch therapy was performed in 45.5% (82) of cases. The median LOS was 3 days, with a cure rate of 95.5% (95% CI: 97-100%). Switch therapy was significantly associated with shorter LOS and substantially lower treatment costs. The average cost per pneumonia patient management was 14363 ± 782 INR ($172.26 ± 9).

**Conclusion:**

Hospital management of pediatric pneumonia was more influenced by early discharge policies than by clinical variables.

**Disclosures:**

**All Authors**: No reported disclosures

